# Protocol for an observational cohort study on psychological, addictive, lifestyle behavior and highly prevalent affective disorders in primary health care adults

**DOI:** 10.3389/fpsyt.2023.1121389

**Published:** 2023-06-09

**Authors:** Fátima Méndez-López, Bárbara Oliván-Blázquez, Marta Domínguez-García, Cruz Bartolomé-Moreno, Isabel Rabanaque, Rosa Magallón-Botaya

**Affiliations:** ^1^Aragonese Primary Care Research Group, Health Research Institute of Aragon (IISA), Zaragoza, Spain; ^2^Network for Research on Chronicity, Primary Care, and Health Promotion (RICAPPS) RD21/0016/0001, Zaragoza, Spain; ^3^Department of Psychology and Sociology, University of Zaragoza, Zaragoza, Spain; ^4^Aragonese Healthcare Service (SALUD), Zaragoza, Spain; ^5^Department of Family and Community Care Teaching - Sector I, Aragonese Healthcare Service, Zaragoza, Spain; ^6^Department of Geography and Territorial Planning, University of Zaragoza, Zaragoza, Spain; ^7^Department of Medicine, Psychiatry and Dermatology, University of Zaragoza, Zaragoza, Spain

**Keywords:** behavior and behavior mechanism, anxiety, depression, mental health, primary health care

## Abstract

**Background:**

Depression and anxious symptoms are prevalent in the general population, and their onset and persistence may be linked to biological and psychosocial factors, many of which are lifestyle-related. The way we manage our care, physical and emotional health and/or discomfort is highly influenced by our own abilities, skills and attitudes despite life’s circumstances. The main aim of this protocol to analyze the relationship between psychological constructs (self-efficacy, activation, health literacy, resilience, personality traits, sense of coherence, self-esteem), and the presence of affective-emotional problems (anxiety, depression) and addictions in primary health care.

**Methods:**

This is a protocol of a prospective longitudinal cohort study including people of 35–74 years old of Aragon primary health care centers (Spain). Three evaluations will be conducted: baseline evaluation, and follow-up assessments five and ten years after recruitment. The primary outcomes will be severity of depression, severity of anxiety, and addictive behaviors. A detailed set of secondary outcomes will be assessed across all three assessments. This will include psychosocial or personal factors on health behavior, social support, lifestyle patterns, quality of life, the use of health and social resources, and chronic comorbid pathology.

**Discussion:**

The analysis of the impact of psychological constructs and lifestyles on the mental health of people and communities will provide evidence that will make it possible to better address and prevent these prevalent problems and address their improvement from a more global and holistic perspective. The evaluation of psychological constructs should be incorporated into health services to improve people’s ability their self-care, the level of knowledge of managing their disease and their physical, mental and social health.

**Clinical trial registration:**

https://www.isrctn.com/, identifier ISRCTN12820058.

## 1. Introduction

Affective disorders are a relevant and growing health problem, with a significant level of morbidity worldwide (1). In 2017, the World Health Organization reported that 4.4% of the world’s population suffered from depression and 3.6% from anxiety. These disorders are more prevalent in women than in men (depression: 5.1% vs. 3.6; anxiety 4.6% vs. 2.6%, respectively) (2). Currently, the COVID-19 pandemic has had a severe impact on the mental health and wellbeing of the world population, increasing cases of major depression by 27.6% and cases of anxiety disorders by 25.6% (3, 4). In Spanish primary health care (PHC), the most frequently registered mental health problems are anxiety disorder (6.7%), and depressive disorder (4.1%) (5). Addictive behaviors are another relevant mental health problem in PHC (6). 33.1% of the population aged 15–64 years consume tobacco daily, while daily alcohol consumption stands at 9.0% (7). Finally, 3.5% of the same population engages in compulsive use of the Internet and 2.8% consumes cannabis daily (7, 8).

According to the International Classification of Diseases 11th, depression, anxiety, and addictive behavior disorders are mental, behavioral, or neurodevelopmental disorders (9). These syndromes are characterized by a clinically significant alteration in the cognition, emotional regulation, or behavior of an individual (9). Moreover, they are the result of complex interactions between social, psychological, and biological factors and are generally associated with significant distress or impairment in personal, family, social, educational, occupational, or other important areas of functioning (10). It must be considered that any mental health problem is a serious health problem when it is of long duration, of at least moderate-severe intensity, and alters the conciliation and daily life (11, 12).

The way we manage our care, physical and emotional health and/or discomfort is highly influenced by our own abilities, skills and attitudes regardless of life circumstances (13, 14). Some of these factors are health literacy (15), patient activation (16), self-efficacy (17), resilience (18), sense of coherence (19), self-esteem (20), and personality characteristics (21). First, the level of health literacy is defined as the knowledge of the population, their motivation and individual abilities to understand and make decisions related to the promotion and management of their health (15). In recent evidence, health literacy interventions improve the emotional state of patients in primary care, with a moderately positive effect on reducing depression and anxiety symptoms (22). Second, self-efficacy is represented as a feeling of confidence in one’s abilities to deal with certain stressors in life (17). Increased self-efficacy beliefs have also been connected to improved emotion management and psychosocial functioning (23, 24). Moreover, low self-efficacy is related, for example, to depression, alcohol use and internet addiction (25–27). Third, resilience represents as a positive adaptation to adversity, such as misfortunes and adverse life events (18). Low resilience has been associated with high levels of depression, addictive behavior, anxiety, and mortality (28–30). Encouraging high resilience in subjects has been found to be an effective method to minimize addictive Internet usage (31, 32). Fourth, sense of coherence is defined as the personal attitudes to the values of vital experiences (19). Some data show a positive relationship between an improved sense of coherence and lower levels of depression, anxiety, and problematic use of the Internet (33, 34). Fifth, self-esteem is represented as a positive or negative feeling about oneself and is constructed through the evaluation of one’s own attributes (20). Adults with higher overall self-esteem are more likely to experience physical, mental, professional and social well-being. For example, low self-esteem is associated with behavioral addictions such as emotional problems, substance abuse, and problematic use of new technologies (35, 36). Finally, the personality traits that influence mental health and addiction include introversion, low conscientiousness, neuroticism, low agreeableness, and low openness (37). Those with much higher neuroticism scores and lower extraversion and conscientiousness scores had higher anxiety and depressive symptoms (38). Addiction to new technologies is associated with adults with personality traits such as neuroticism, openness, and low conscientiousness (39, 40).

Furthermore, the onset and persistence of affective disorders has been associated with specific lifestyle characteristics (e.g., poor-quality diet, sleep disturbances, and sedentary lifestyle) (41–43). Therefore, some healthy habits (e.g., good nutrition, quality sleep, sufficient physical activity) are associated with lower levels of depression and anxiety (44, 45). In particular, improving physical activity has a moderate to large effect on improving depressive symptoms (46). Additionally, depression severity and current depression diagnosis are associated with unhealthy dietary intake and poorer food quality (47). Moreover, sleep disturbances increase the risk of suicidal behavior in depressed patients (48). Furthermore, addictive behaviors (e.g., tobacco use, harmful use of alcohol, substance abuse, and compulsive use of the Information and Communications Technologies (ICT) can coincide with, contribute to, or result from mental disorders such as depression or anxiety (49–51). Current evidence suggests the high probability of exacerbation of anxious or depressive disorders when they coexist with chronic comorbidities (52). Several studies have found that people with chronic diseases have a higher risk of developing mental disorders such as depression or anxiety (52–54). Simultaneously, people diagnosed with a mental health disorder are more likely to have chronic physical conditions than a person without a mental health diagnosis (52, 55). Moreover, a prospective cohort study of community individuals with a follow-up after 11 years indicated that the increase in depressive symptoms over time was associated with higher mortality rates (56).

Mental health disorders generate a great economic burden in the use of health system services and represent a significant proportion of health-seeking contacts in primary care (57, 58). In Spain, around 70–85% of the population opts for public primary health care over private insurance (59). Specifically, 60% of frequent users in primary health care suffer from some mental health problem, particularly depressive or anxiety disorders (60). Only 10% of patients with mental health problems seen in primary care are referred to mental health units (61). A World Health Organization report highlights the fundamental role of PHC personnel in the detection, diagnosis, and treatment of people with mental disorders (62, 63). The importance of the PHC lies in being the gateway to the health care system, its accessibility and its interaction with people with mental health problems and their families (61). Most people with mental health problems access primary care, and their disorders are more likely to be identified and treated appropriately and with less risk of stigma (62). In addition, the integration of mental health into primary care promotes comprehensive, coordinated, and person-cantered care for the many people with co-morbid physical and mental health problems (62).

Promoting the participation of the population in coping with diseases and their self-care and self-management of health is a key element in the health of the population in general and especially of the population with affective disorders (64). It is necessary to investigate further how people’s psychological constructs, beliefs and self-care capacity, as well as their lifestyles, can affect the well-being or discomfort of people and contribute to a healthier life from the perspective of mental health. Furthermore, it is important to assess longitudinal changes in these factors and affective disorders given their association with increased comorbidity and mortality (56). The analysis of the impact of psychological constructs and lifestyles on the mental health of individuals and communities will provide evidence that will make it possible to better address and prevent these prevalent problems as well as foster their improvement from a more global and holistic perspective.

Our main objective in this study is to analyze the relationship between psychological constructs (self-efficacy, activation, health literacy, resilience, personality traits, sense of coherence, self-esteem), affective-emotional problems (anxiety, depression) and addiction in primary health care. Therefore, our main hypothesis is that subjects with lower levels of the psychological constructs (self-efficacy, activation, health literacy, resilience, sense of coherence, self-esteem) will have more severe depressive, anxious, and addictive behavior symptoms than those with higher values of the psychological constructs.

One of the secondary objectives of this study is to analyze whether these psychological constructs act as mediating or moderating factors in the relationship between the diagnosis of comorbidities or healthy lifestyles and depression, anxiety, and addictive disorders. Therefore, our secondary hypothesis is that the relationship between the diagnosis of comorbidities or healthy lifestyles and depression, anxiety, or addictive disorders will change according to the value of the different moderators, such as self-efficacy, activation, health literacy, resilience, personality traits, sense of coherence, and self-esteem.

Moreover, a final secondary objective is to create a cohort for an analysis of the relationship between these psychological constructs and lifestyles and the incidence rate of diagnoses of depression and/or anxiety to be revisited after 5–10 years. Consequently, our last secondary hypothesis is that people with lower levels of the psychological constructs and less healthy lifestyle habits will be more likely to experience increased incidence of diagnoses of depression and anxiety or worsening symptoms in the following 5 to 10 years than will those with higher values of the psychological constructs of healthy lifestyles.

## 2. Materials and methods

### 2.1. Study design

This study is a protocol of a prospective longitudinal cohort study. This study will be the first baseline measurement for creating a cohort with expected follow-up at 5–10 years. According to current evidence from prospective studies in the community population (56), and the advantages of a cohort study, follow-up at 5–10 years will make it possible to determine incidence, natural history, clinical course, and assess mortality (65). This protocol was registered with ISRCTN Registry before commencement (ISRCTN12820058). For the creation of this protocol, we followed the standard protocol items: recommendations for interventional trials (SPIRIT) guidance (Supplementary Material 1), and the study will be performed and reported according to the guidance for strengthening the reporting of observational studies in epidemiology (STROBE).

### 2.2. Contextual framework

The study will be carried out within the framework of primary health care in the Spanish region of Aragon. Aragon is an Autonomous Community located in the northeast of Spain. It is the fourth Spanish Autonomous Community by territory but ranks 11 out of 17 in terms of population. It has a population of 1.3 million inhabitants: 60% live in urban areas (675,301 inhabitants live in the city of Zaragoza) and semi-urban areas and 40% of the remaining population lives in towns with fewer than 5,000 inhabitants. Aragon has an aging population, with 23.5% of people over 64 years of age. The average socioeconomic level of Aragon is medium, and the unemployment rate is lower than the national average. Public health provides assistance for nearly the entire population [around 70–85% of the population opts for public primary health care over private (59)]. Aragon public primary health care is structured into 8 health sectors organized into 123 Basic Healthcare Areas (BHA), which include 118 health centers and 870 local clinics (66, 67). Spanish primary health care is made up of a multidisciplinary team of general practitioners, nurses, psychologists, social workers, physiotherapists and occupational therapists.

### 2.3. Inclusion and exclusion criteria

The inclusion criteria are (A) participants of 35–74 years old; (B) ability to speak and write in Spanish; (C) ability to understand the study and provide consent to participate in the study voluntarily. According to national clinical data from Primary Care, the most frequent stage of onset of depression and anxiety is the decade between 30 and 40 years of age. At this age, these disorders are more prevalent in women than in men (depression: 4.2% vs. 2.0%; anxiety: 10% vs. 5.7%, respectively) (5). In addition, in this age range, these affective disorders have a notable impact on people who are usually at the age of full work, economic and family activity (68). The prevalence of these pathologies increases with age, decreasing slightly from the age of 75, and the trend of higher prevalence in women than in men continues to increase with age (depression: 12.2% vs. 4.8%; anxiety: 11.3% vs. 5.0%, respectively) (5).

The exclusion criteria are (A) suffering from a terminal illness; (B) being institutionalized at the time of the appointment; (C) difficulty participating due to cognitive dysfunction, dementia, or any serious disease that may seriously interfere with the patient’s participation in the study; (D) persons who plan to move out of Spain within five years after the initiation of the study.

### 2.4. Sample size and sampling

The sample size was computed using the formula for caclulating the population estimates of the most prevalent affective disorders in primary care (anxiety and depression) (69). To calculate the sample size, we will use the data obtained in the study by Santomauro et al. (57). We used the prevalence of the most frequent affective disorders and addictions in primary care (anxiety, depression) as the main variable: 3.1% for major depression and 4.8% for anxiety disorders. Accepting an alpha value of 0.05 and a margin of error of 3% of units, the total sample size required was 290 with the expectation of a possible 30% withdrawal rate.


n=Zα2⁢p⁢(1-p)e2

**FIGURE 1:**
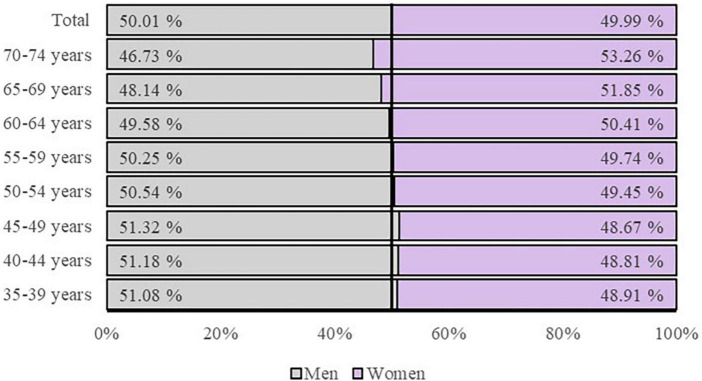
Total population distribution of Aragon by age and sex respect to National Institute of Statistics data from the census of Aragon 2021.

For selecting potential participants, stratified selection will be made by age, sex and population distribution in urban and rural areas with respect to the National Institute of Statistics data from the census of Aragon 2021 (Figure 1 and Table 1). This stratification will be carried out with the intention of establishing the greatest variability within the sample and reaching a maximum level of representativeness for the population (Table 2). To achieve this, different primary care health centers in the autonomous community, 1–2 rural centers and 1–2 urban centers, will be selected. The rural centers sampled will be in towns with a population of under 2,000 inhabitants.

**TABLE 1:** Total population distribution in urban and rural areas of Aragon respect to National Institute of Statistics data from the census of Aragon 2021.

Province	Urban area, *n* (%)	Rural area, *n* (%)
Teruel	36,240 (27.01)	97,936 (72.99)
Zaragoza	701,969 (72.18)	270,559 (27.82)
Huesca	53,956 (24.23)	168,731 (75.77)
Total	792,165 (59.59)	537,226 (40.41)

**TABLE 2:** Stratified selection of Aragon population by sex, age, and population distribution.

Years	Total			Rural (40.41%)			Urban (59.59%)		
	**Both**	**Men**	**Women**	**Both**	**Men**	**Women**	**Both**	**Men**	**Women**
35–39	49	25	24	20	10	10	29	15	14
40–44	59	30	29	24	12	12	35	18	17
45–49	58	30	28	23	12	11	35	18	17
50–54	55	28	27	22	11	11	33	17	16
55–59	52	26	26	21	11	10	32	16	16
60–64	46	23	23	18	9	9	28	14	14
65–69	39	19	20	15	7	8	23	11	12
70–74	36	17	19	18	7	8	21	10	11
Total	395	198	197	158	79	79	237	119	118

### 2.5. Data collection

The research team will perform an information session about the study at the selected health centers. In these sessions, the research team will explain the project to be carried out and its methodology to all primary care professionals (family doctors, nurses, psychologists, social workers, physiotherapists) who work in the selected health centers. With these informative sessions for PHC professionals and a good line of contact established with the research team, the aim is to ensure and improve the recruitment rate since PHC professionals maintain good and trustworthy contact with the population. They will also be provided with the telephone number of the research unit for the initial appointment if any patient that these professionals receive in their daily work is interested in participating in the study.

The research team will send an information letter to the participants obtained from the stratified sampling. This letter will include information on the study procedure and contact details of the research unit, so participants who are interested can contact the research team. All members of the multidisciplinary research group come from primary health care and public health.

**FIGURE 2:**
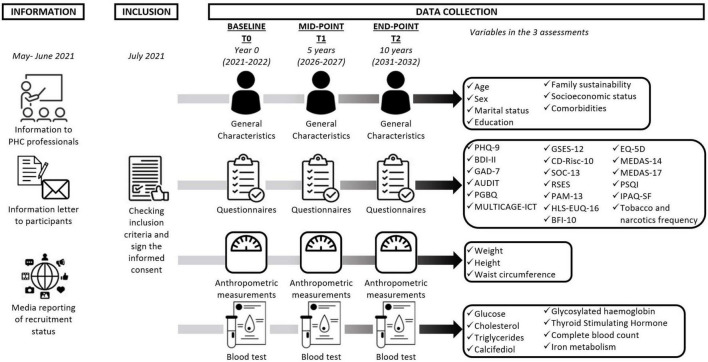
Chronological data collection. PHQ-9, Patient Health Questionnaire; BDI-II, Beck Depression Inventory; GAD-7, Generalized Anxiety Disorder; AUDIT, Alcohol Use Disorders Identification Test; PGBQ, Pathological Gambling Brief Questionnaire; MULTICAGE-ICT, MULTICAGE-Information and Communications Technologies; GSES-12, General Self Efficacy Scale; CD-Risc-10, Connor–Davidson Resilience Scale; SOC-13, Sense of Coherence Scale; RSES, Rosenberg Self-Esteem Scale; PAM-13, Patient Activation Questionnaire; HLS-EUQ-16, Health Literacy Survey European Questionnaire; BFI-10, Big Five Inventory; EQ-5D, European Quality of Life-5 Dimensions Questionnaire; MEDAS, Mediterranean Diet Adherence Screener; PSQI, Pittsburgh Sleep Quality Index; IPAQ-SF, International Physical Activity Questionnaire-Short Form.

In addition, broadcasting will be carried out through general media, posters, and press releases, which will raise awareness and obtain greater data at the beginning of the study. Patients who will be interested in the study will arrange a meeting with the research team at their health center to explain the study in more detail, verify that they meet the inclusion and stratification criteria and sign the informed consent if they wish (Supplementary Material 2). All researchers will be trained to conduct research in a systematic and unified method for data collection. A research assistant will collect the data, and another will enter and encode the data into a database. All subjects’ data will be anonymized and will only be used for the purposes of the study. To maintain anonymity, researchers who manage the data of the database or who perform the evaluation of the results and the analysis of the data will not know or have access to data that would allow them to identify the patient directly. At each appointment, the different questionnaires will be administered, a physical examination will be performed that will include anthropometric measurements, and a second appointment will be made with the participant to perform a blood draw at their health center that same week. The research team will include a monitoring and management committee. It will review the quality of the data included in the database and will discuss any conclusions resulting from data of doubtful origin. Additionally, this committee will monitor recruitment rates, dropout rates, and any concerns related to the study. The reasons for dropping out, having made the appointment, or even the reasons for definitive non-participation will also be recorded. Participants and health professionals will be informed about these results. Figure 2 details the registration and evaluation schedule for this study, with dates included.

### 2.6. Measures

An evaluation will be carried out at baseline with further assessments five and ten years following the end of the baseline assessment. In this study, we will use valid instruments whose psychometric properties have been tested on the Spanish population to collect data; the validity and reliability of the questionnaires are reported in Table 3.

**TABLE 3:** Instruments used in the study.

	Instrument	Developed by	Translated by	Item number	Range of score	Higher scores mean	Reliability developer	Translator
Primary outcomes	PHQ-9	Kroenke and Spitzer (70)	Muñoz-Navarro et al. (71)	9 (4-point Likert 0-3)	0–27	Depression ↑	α = 0.89	α = 0.80
	BDI-II	Beck et al. (72)	Sanz et al. (73)	21 (4-point Likert 0-3)	0–63	Depression ↑	α = 0.91	α = 0.87
	GAD-7	Spitzer et al. (74)	García-Campayo et al. (75)	7 (4-point Likert 0-3)	0–21	Anxiety ↑	α = 0.92	α = 0.93
	AUDIT	Saunders et al. (76)		10 (5-point Likert 0-4)	0–40	Alcohol problem↑	α = 0.93	
	PGBQ	Fernández-Montalvo et al. (77)		4 (Dichotomous 0-1)	0–4	Gambling addiction ↑	α = 0.95	
	MULTICAGE -ICT	Pedrero Pérez et al. (51)		20 (Dichotomous 0-1)	0–4 (Each factor)	ICT compulsive behaviors ↑	α = 0.93	
	GSES-12	Bosscher et al. (78)	Herrero et al. (79)	12 (5-point Likert 1-5)	12–60	Self-efficacy ↑	α = 0.74	α = 0.69
Secondary outcomes	CD-Risc-10	Campbell-Sills and Stein (18)	Notario-Pacheco et al. (80)	10 (5-point Likert 0-4)	0–40	Resilience ↑	α = 0.85	α = 0.85
	SOC-13	Antonovsky (19)	Moreno et al. (81)	13 (7-point Likert 1-7)	13–91	Sense of coherence ↑	α = 0.91	α = 0.83
	RSES	Rosenberg (20)	Martín-Albo et al. (82)	10 (5-point Likert 1-5)	10–50	Self-esteem ↑	α = 0.77	α = 0.85
	PAM-13	Hibbard et al. (16)	Moreno-Chico et al. (83)	13 (4-point Likert 1-4)	13–52	Patient activation ↑	α = 0.87	α = 0.7
	HLS-EUQ16	Sørensen et al. (15)	Nolasco et al. (84)	16 (Dichotomous 0-1)	0–16	Health literacy ↑	α = 0.91	α = 0.98
	BFI-10	John et al. (21)	Benet-Martínez and John (37)	10 (5-point Likert 1-5)	2–10 (Each factor)	Extraversion, Agreeableness, Neuroticism Conscientiousness, Openness↑	α = 0.83	α = 0.78
	EQ-5D	The EuroQol Group et al. (85)	Badia et al. (86)	5 (3-point Likert 1-3)		Better quality of life ↓	ρ = 0.99	α = 0.78
	MEDAS-14	Schröder et al. (87)		14 (Dichotomous 0-1)	0–14	Adherence to the Mediterranean diet ↑	*k* = 0.43 *r* = 0.52	
	MEDAS-17	Schröder et al. (88)		17 (Dichotomous 0-1)	0–17	Adherence to the Mediterranean diet ↑	*k* = 0.41 *r* = 0.61	
	PSQI	Buysse et al. (89)	Royuela-Rico and Macías-Fernández (90)	19 (4-point Likert 0-3)	0–21	Sleep quality↓	α = 0.83	α = 0.81
	IPAQ-SF	Craig et al. (91)	Puig-Ribera et al. (92)	7 (days and minutes of activity)	MET-minutes/we	Physical activity ↑	ρ = 0.96	*k* = 0.70

↑, going up; ↓, going down; α, Cronbach’s alpha; ρ, Spearman’s rho; k, kappa test-retest reliability; PHQ, Patient Health Questionnaire; BDI, Beck Depression Inventory; GAD, Generalized Anxiety Disorder; AUDIT, Alcohol Use Disorders Identification Test; PGBQ, Pathological Gambling Brief Questionnaire; ICT, Information and Communications Technologies; GSES, General Self Efficacy Scale; CD-Risc, Connor–Davidson Resilience Scale; SOC, Sense of Coherence Scale; RSES, Rosenberg Self-Esteem Scale; PAM, Patient Activation Questionnaire; HLS-EUQ, Health Literacy Survey European Questionnaire; BFI-10, Big Five Inventory; EQ-5D, European Quality of Life-5 Dimensions Questionnaire; MEDAS, Mediterranean Diet Adherence Screener; PSQI, Pittsburgh Sleep Quality Index; IPAQ-SF, International Physical Activity Questionnaire-Short Form.

#### 2.6.1. Primary outcomes

The main variables will be severity of depression, severity of anxiety, and addictive behaviors.

##### 2.6.1.1. Depression

Depression will be assessed by Patient Health Questionnaire-9 and Beck Depression Inventory. The Patient Health Questionnaire-9 (PHQ-9, Spanish version) will be used to measure the degree of depression in the past 2 weeks (70). The severity levels include no depression (0–4), mild depression (5–9), moderate depression (10–14), moderately severe depression (15–19) and severe depression (20–27). The validated Spanish version has a Cronbach’s alpha value (α) of 0.80 (71). The Beck Depression Inventory-Second Edition (BDI-II, Spanish version) will be used to measure the severity of depression (72). The severity levels include minimal depression/no depression (0–13), mild depression (14–19), moderate depression (20–28), and severe depression (29–63). In its Spanish version, the Cronbach’s alpha coefficient is 0.87 (73).

##### 2.6.1.2. Anxiety

The Generalized Anxiety Disorder-7 (GAD-7, Spanish version) will be used to measure anxiety level. Each item describes one of the typical symptoms of generalized anxiety disorders experienced over the last 2 weeks (74). The severity levels in the original study include no anxiety (0–4), mild anxiety (5–9), moderate anxiety (10–14) and severe anxiety (15–21). The validated Spanish version has a Cronbach’s alpha value (α) of 0.93 (75).

##### 2.6.1.3. Alcohol consumption

The Alcohol Use Disorders Identification Test Form (AUDIT, Spanish version) will be used to screen for excessive alcohol consumption. It will act as a support in the evaluation and identification of excessive alcohol consumption as a cause of disease, dependence and consequences of harmful consumption (76). Alcohol consumption dependence levels include no dependence (0–7), low dependence (8–15), moderate dependence (16–19) and high dependence (20–40). The validated Spanish version has a Cronbach’s alpha value (a) of 0.93 (75).

##### 2.6.1.4. Pathological gambling

The Pathological Gambling Brief Questionnaire (PGBQ) will be used to assess the psychometric behavior with respect to gambling addictions in the general population (77). A score ≥ 2 indicates possible gambling addiction problems, with a maximum score of 4. In its Spanish version, the Cronbach’s alpha coefficient is 0.95 (77).

##### 2.6.1.5. Pathological use of information and communication technologies

The MULTICAGE-ICT Questionnaire will be used to Assess problems related to the use of the Internet, mobile phones, video games, instant messaging, and social networks (51). Composed of 20 total items distributed in 5 scales (internet; mobile phone; video games; instant messaging; social networks). In each scale, item 1, the estimation of excess in temporary dedication; item 2, the estimation of significant others; item 3, the difficulty of not performing the behavior; item 4, the difficulties in voluntarily interrupting the behavior. Higher scores indicate higher use/abuse of Information and Communications Technologies (ICT). The validated Spanish version has a Cronbach’s alpha value (α) of 0.93 (51).

#### 2.6.2. Secondary outcomes

##### 2.6.2.1. Sociodemographic and clinical data

Sociodemographic information, including age, sex, marital status, education, socioeconomic status, and family sustainability, will also be obtained through an *ad hoc* questionnaire prepared by the research team. Health-related characteristics will include physical and psychological comorbidities. These data will be collected from the electronic medical record and will be included in the database following the definitions of diseases according to the International Classification of Primary Care (3rd edition). In addition, the physical examination will obtain anthropometric measurements (weight, height, and waist circumference). The analytical determination will complement the diagnosis of comorbidities with chronic diseases. In all patients, the concentrations of glucose (mg/dL), cholesterol, triglycerides, glycosylated hemoglobin (%), Thyroid-Stimulating Hormone (μIU/mL), complete blood count, iron metabolism, and calcifediol will be obtained.

##### 2.6.2.2. Self-efficacy

The General Self Efficacy Scale (GSES-12, Spanish version) will be used to evaluate perceived global self-efficacy as feeling confident in one’s abilities to adequately handle certain stressors in life (78). The final score on the questionnaire is the sum of the responses obtained on each item (range 12–60) and higher scores indicate higher levels of self-efficacy. The validated Spanish version has a Cronbach’s alpha value (α) of 0.69 (79).

##### 2.6.2.3. Resilience

The Connor–Davidson Resilience Scale (CD-Risc-10, Spanish version) will be used to evaluate resilience as the positive adaptation to circumstances of significant adversity such as misfortunes and tragic situations in life (18). The final score on the questionnaire is the sum of the responses obtained on each item (range 0–40), and higher scores indicate higher levels of resilience. The validated Spanish version has a Cronbach’s alpha value (α) of 0.85 (80).

##### 2.6.2.4. Sense of coherence

The Sense of Coherence Scale (SOC-13, Spanish version) will be used to measure sense of coherence in terms of the personal disposition toward the values of vital experiences. It also measures understandability, manageability and meaning (19). Higher scores (after flipping the flipped items) (range 13–91) indicate a greater sense of coherence. The validated Spanish version has a Cronbach’s alpha value (α) of 0.83 (81).

##### 2.6.2.5. Self-esteem

The Rosenberg Self-Esteem Scale (RSES, Spanish version) will be used to assess self-esteem during the past 2 weeks (20). Self-esteem is defined as feelings toward oneself, which can be positive or negative, and is determined based on an evaluation of one’s own characteristics. Scores below 25 points are considered to reflect significant low self-esteem issues. The validated Spanish version has a Cronbach’s alpha value (α) of 0.85 (82).

##### 2.6.2.6. Patient activation

The Patient Activation Questionnaire (PAM-13, Spanish version) will be used to measure activation as the capacity and ability to manage one’s personal condition of his/her health and how competent he/she feels in taking on this role (16). The final score on the questionnaire is the sum of the responses obtained on each item (range 13–52) and higher scores indicate a higher level of patients’ activation in addressing their own health. We decided not to include the category “not applicable” to avoid misinterpretations. In its Spanish version, the Cronbach’s alpha coefficient is 0.7 (83).

##### 2.6.2.7. Health literacy

The Health Literacy Survey European Questionnaire (HLS-EU-Q16, Spanish version) will be used to measure the level of health literacy as knowledge of the population, motivation, and individual abilities to understand and make decisions related to the promotion and management of participants’ own health (15). The score of each subject will be obtained from the sum of the scores of the 16 items, transforming each one into a dichotomous response: very difficult and difficult = 0; easy and very easy = 1. The health literacy levels include an inadequate or problematic level (0–12) and a sufficient level (13–16). In its Spanish version, the Cronbach’s alpha coefficient is 0.98 (84).

##### 2.6.2.8. Personality characteristics

The Big Five Inventory-10 (BFI-10, Spanish version) will be used to examine personality characteristics (21). The questionnaire measures the 5 factors of personality from the five factors model (each factor entails two items). Extraversion (Extraverts engage actively with others to earn friendship, admiration, power, status, excitement, and romance; Introverts conserve their energy and do not work as hard to earn these social rewards). Agreeableness (High scores have a great deal of empathy and tend to get pleasure out of serving and taking care of others; Low scorers are often described as hostile, competitive, and antagonistic). Conscientiousness (High scorers are organized and determined; Low scorers are impulsive and easily side-tracked). Neuroticism (High scorers are more likely to react to a situation with fear, anger, sadness, and the like. Low scorers are more likely to brush off their misfortune and move on). Openness (High scorers tend to be creative, adventurous, and intellectual; Low scorers tend to be practical, conventional, and focused on concrete information). The validated Spanish version has a Cronbach’s alpha value (α) of 0.78 (37).

##### 2.6.2.9. Health-related quality of life

The European Quality of Life-5 Dimensions Questionnaire (EQ-5D, Spanish version) will be used to measure health-related quality of life. It will be used to calculate the quality-adjusted life year (QALY) during the monitoring period by adjusting the length of time affected by the health result in relation to the utility value (85). It contains five health dimensions (mobility, self-care, usual activities, pain/discomfort and anxiety/depression), and each of these has three levels (no problems, slight or moderate problems, and severe problems). Moreover, this questionnaire incorporates a Visual Analog Scale (VAS) which can be used as a quantitative measure of health outcome reflecting the patient’s own judgment. Patients mark the point on the vertical line that best reflects their assessment of their current global health status. In its Spanish version, the Cronbach’s alpha coefficient is 0.78 (86).

##### 2.6.2.10. Diet adherence

The Mediterranean Diet Adherence Screener (MEDAS) was developed within the Mediterranean diet (PREDIMED) study group. We will also evaluate the adherence to the Mediterranean diet, assessed with the MEDAS (14 items) (87) and MEDAS plus (17 items) (88) questionnaires. Both questionnaires share many of their items, including food and consumption habits: the use of olive oil as the main source of cooking fat, preference for white meat over red meat, servings of vegetables, portions of fruit, red meat or sausages, servings of animal fat, sugar-sweetened beverages, red wine, legumes, fish, commercial pastries and dressing foods with a traditional sauce made of tomatoes, garlic, onion or leeks sautéed in olive oil. However, the stricter values of some items and the inclusion of some additional items in the 17-point version is an attempt to better reflect the possible caloric restriction that should be applied to the Mediterranean diet pattern when the goal is to lose weight. The MEDAS-14 levels include low adherence (0–8) and good adherence (9–14) (87). The MEDAS-17 levels include low adherence (0–7), medium adherence (8–10) and high adherence (11–17) (88). The construct validity of MEDAS was determined by analyzing the correlations of the MEDAS score with dietary intake reported on the food frequency questionnaire (FFQ) with a moderate correlation and moderate mean agreement for both versions (*r* = 0.52 and *k* = 0.43 for the 14-item version and *r* = 0.61 and *k* = 0.41 for the 17-item version) (87, 88).

##### 2.6.2.11. Sleep quality

Pittsburgh Sleep Quality Index (PSQI, Spanish version) will be used to measure quality and patterns of sleep over the past month (89). It differentiates between “poor” and “good” sleep by measuring seven domains: subjective sleep quality, sleep latency, sleep duration, habitual sleep efficiency, sleep disturbances, use of sleep medication and daytime dysfunction. It consists of 19 self-applied questions and five questions that request the evaluation of the patient’s bedmate or roommate (these are not scored). The sleep quality levels include good sleep quality (0–5) and poor sleep quality (6–21). The validated Spanish version has a Cronbach’s alpha value (α) of 0.81 (90).

##### 2.6.2.12. Physical activity

International Physical Activity Questionnaire-Short Form (IPAQ-SF, Spanish version) will be used to measure levels of habitual physical activity over the last 7 days (91). It has seven items and records the activity of four intensity levels: vigorous-intensity activity, moderate-intensity activity, walking and sitting. A total physical activity result MET (minutes/week) can be calculated as the following: total physical activity is the sum of the total results (walking + moderate + vigorous) MET. The Physical Activity levels include low (0-600 MET), moderate (601-3000 MET), high (more than 3000 MET). The validated Spanish version has a kappa value (k) of 0.70 (92).

##### 2.6.2.13. Consumption of tobacco and narcotic substances

Examination of the frequency of consumption of substances such as tobacco and narcotics will be carried out through an *ad hoc* questionnaire prepared by the research team measured with the 4-question scale adapted from the WHO MONICA study (91, 92). It will assess the current consumption or time since the last consumption, starting age, type of substance consumed and the specific amount of each substance.

### 2.7. Data analysis

Baseline analysis of the results: Descriptive analysis of all variables (percentage and confidence interval for qualitative variables; means and standard deviation for parametric quantitative variables or median and interquartile range for non-parametric quantitative variables) and univariate analysis (we will use the T-Student for parametric quantitative variable, Mann-Whitney U for non-parametric quantitative variables, and the Chi-Square test for qualitative variables) to evaluate the differences between the patients who have some of the most prevalent affective-emotional problems in primary care (anxiety, depression) compared with those who do not. Statistical analyses will be chosen based on the size of the sample (parametric or non-parametric tests). To compare the two groups, missing data will be assessed and the need to use an intention-to-treat (ITT) analysis and a multiple imputation (MI) technique to handle missing data will be assessed.

To answer the main objective, we will use a multiple linear regression model. To do this, each main variable, the PHQ-9 score, BDI-II, GAD-7, PGBQ, AUDIT, and MULTICAGE-ICT, will be used as a continuous variable. Multiple linear regression will be performed using a stepwise method to obtain a better fit result to the statistical analysis. This stepwise regression simply will repeat the multiple regression, each time removing the least correlated variable. Only the significant variables obtained in the bivariate analysis will be entered into the regression model.

Finally, mediation/moderation analyses will be carried out to analyze whether psychological constructs participate as a mediator or as a moderator in the association of lifestyles (exposure) and anxiety, depression and/or addictions (outcome). The PROCESS analytical tool developed by Hayes will be used to assess mediation/moderation. These analyses were based on multiple linear regression path analysis (93). Bootstrap resampling (10.000 samples) will be used to estimate 95% confidence intervals. Given that heteroscedasticity is common in cross-sectional data, all analyses will include a correction for heteroscedasticity (HC0) (94). The Johnson-Neyman technique will be used to compute the range of significance and simple slopes for the interaction analyses (93). We will report unstandardized regression coefficients; all analyses will be two-tailed and used conventional significance thresholds (α = 0.05). Data collection and statistical analysis will be performed using Excel software, SPSS software (version 25.0) (95) and the statistical software environment R (version 3.6.2) (96).

## 3. Discussion

Affective disorders are a serious and expanding public health issue, with a high morbidity rate worldwide (57, 58). According to the Global Burden of Disease Study (GBD), depression is the third cause in women and the fifth in men of years lived with disability (97). 60% of frequent users in primary health care suffer from a depressive or anxiety disorder (60). However, only 9% of all primary care patients with depression and anxiety receive adequate treatment and only 6% achieve remission, making affective disorders a significant management issue in primary care (98). Addictive behaviors are another relevant mental health problem in PHC (6). The harmful use of alcohol and tobacco or other substances has serious repercussions on public health and is considered one of the main risk factors for poor health worldwide (99). Not only the consumption of addictive substances is relevant. In recent years, there has been a growing convergence between gaming and betting on various platforms, aided significantly by the internet (100).

The onset and persistence of affective disorders have been associated with psychological constructs (self-efficacy, activation, health literacy, resilience, personality traits, sense of coherence, self-esteem) and lifestyles (poor-quality diet, sleep disturbances, and sedentary lifestyle, as well as alcohol consumption, tobacco, and other addictions) (43). These psychological constructs are framed around the theory of salutogenesis (14). The salutogenic approach seeks to improve participants’ mental health and well-being by enhancing their knowledge, confidence, and ability to employ personal health-related elements (101).

There is evidence from longitudinal studies that evaluate personal factors in the adult population and their relationship with the development of depressive and anxious symptoms. In several cohort studies in older patients, advancing age, alcohol use, sleep problems, severe pain, and multiple disease burden are shown to be risk factors for predicting the development of depression and anxiety in participants who did not have these mental health problems at baseline. Protective factors were income, social support, higher self-efficacy, resilience, and well-being at baseline (102, 103). In another general population study with the same age as our study population, it was shown that individuals who showed higher self-esteem and cognitive ability were negatively associated with a latent initial level of depression (104). These studies support the potential feasibility of this study. The analysis of different psychological constructs in a general population can provide us with an interesting perspective on their relationship to the development and persistence of affective problems about which there is not much research.

Primary health care is the ideal setting for a psychosocial conflict and mental illness prevention approach for three reasons (105). First, PHC is perhaps the social instrument with which the greatest number of citizens are exposed during the year and in the life of each one of them. Second, numerous studies have shown that patients with chronic psychosis or psychosocial conflict often refer to different PHC settings multiple times. Third, at a pragmatic and health level, emotional disorders that are not detected early by primary care providers have a worse prognosis (105). Based on current evidence, a randomized clinical trial conducted in primary care evaluated whether a complex intervention to promote the Mediterranean diet, physical activity, and/or smoking cessation is effective in preventing depression at 12 months follow-up of a population aged 457–75 years. As a result, the intervention provided a non-significant reduction in the incidence of major depression, compared with usual care (106). Our study will provide the opportunity to evaluate and explore what individual conditions influence the evolution of the disease or the development of new diagnoses of affective-emotional disorders so prevalent in the community, such as depression and anxiety. The findings of our study will allow the development of individualized and preventive interventions to provide timely help to adults in groups at risk of developing one of these mental disorders or to reduce symptoms in those who have already been diagnosed.

### 3.1. Strengths and limitations

The strengths of this study include the design and the wide range of outcome measures. Another strength is that it involves recruiting a general adult population from a Spanish region whose sociodemographic characteristics (age, sex, population distribution) are very similar to those of the rest of the European population and other regions of the world (107). These characteristics will allow a greater representativeness of the results and a better extrapolation of the results to other regions. Public health covers practically the entire population (around 70–85% of the population opts for public primary health care over private) (59). Due to this, a recruitment of the population from PHC will allow us to improve the scope of the representativeness of the sample.

Our study will provide a wealth of information on the interaction between depression, anxiety, addictive behaviors, personal health behavioral factors, and lifestyles. In addition, no longitudinal studies carried out in our population have been identified that analyze in depth all these psychological constructs together while considering their impact on health. Therefore, this study will allow us to provide new evidence on the relationship between psychological constructs and lifestyles in the onset and development of depression and anxiety.

This study includes self-report and hetero-administered questionnaires, as well as a physical and laboratory examination. Among the limitations of the study will be the difficulty of the questionnaires used. This difficulty will be minimized by prior training and quality control of the data collection process to ensure that accurate results comparable to other studies are obtained.

The most important difficulties of the study would be a possible low participation rate and the possible withdrawal of participants due to refusal to complete the follow-up (65). However, possible reasons for dropout and other problems will be recorded. One strategy to achieve a high initial participation rate is to send an informative letter with information about the study; in this way, when the potential participant is contacted, they already know the objective and their confidence in the study is greater. Another of the strategies used for a higher participation rate is the contact of primary care professionals with potential study participants. It has been shown that the general population feels more comfortable when talking to their PHC professionals given their closeness and habitual treatment (108). Due to the possibility of follow-up and continuity of care and attention to the population, PHC is the ideal means to carry out this type of prospective study. The privileged relationships and patient care-oriented knowledge that PHC professionals can establish with patients represents an important tool that could contribute to reducing the global burden of chronic diseases (109). The promotion of the participation of the population in coping with diseases and their self-care and self-management of health is a key element in addressing the health of the general population and especially in the part of the population with mental disorders.

The evaluation of psychological constructs should be incorporated into health services to improve people’s ability to improve their self-care, as well as their level of knowledge of managing their disease and their physical, mental and social health. Mental health is a social and health challenge.

## Ethics statement

This study was approved by the Clinical Research Ethics Committee of Aragon N° PI20/302. This study was developed in accordance with the Declaration of Helsinki. Since the project involves the collection and processing of personal data, including personal information; the collection, treatment, communication, and transfer of personal data of all participating subjects must comply with the provisions of the General Data Protection Regulation (EU) (GDPR 2016/679) and the applicable national legislation, Organic Law 3/2018, of December 5, on the Protection of Personal Data. Informed consent was obtained from all participants. All subjects allowed their data to be anonymized and used only for the purposes and publication of the results of this study.

## Author contributions

RM-B and BO-B conceived the quantitative part of the study. FM-L and RM-B led the drafting of this manuscript. RM-B obtained the ethical approval from each institution and contributed to design the training program on the questionnaires and measurements. CB-M, MD-G, and IR advised and contributed to the study design. FM-L, CB-M, and BO-B developed the statistical analysis plan. All authors reviewed the manuscript content and approved the final version for submission addressed.
