# Supplementary material for: Protocol for an observational cohort study on psychological, addictive, lifestyle behavior and highly prevalent affective disorders in primary health care adults
Source: Front Psychiatry. 2023 Jun 9;14:1121389. doi: 10.3389/fpsyt.2023.1121389 (PMC10288582; doi:10.3389/fpsyt.2023.1121389)
Supplement: Supplementary file 1 [file Data_Sheet_1.docx]

**SUPPLEMENTARY 1: Information and consent document for the participant**

**INFORMATION DOCUMENT FOR THE PARTICIPANT**

**Exploring the relationship between psychological constructs, lifestyles, and highly prevalent affective-emotional and addictive behaviours in a general population in Primary Health Care.**

Dear Sir,

The Research Network on Preventive Activities and Health Promotion includes various research groups that want to generate knowledge to improve control over health in health promotion and disease prevention in Primary Care.

We invite you to participate in this study. Before deciding if you want to participate, it is important that you understand the objectives, the importance of your participation and what it will consist of, as well as how the data collected will be used and the possible benefits and risks.

***Description of the process of your participation***

Prior to your final decision to participate in this study, we explain that the objective of the study is to analyze whether personal skills related to behaviors are related to outcomes in lifestyles, physical health, mental health, quality of life, and use of health services. It is also of interest to study to delve into the opinions and experiences of the population on the relationship between personal skills (self-efficacy, activation, health literacy, resilience, locus of control and personality traits) with their perception of health, their styles of life and their quality of life.

If you have any questions before, during or after the completion of the study, we will answer any questions you may have. Participation in the study is completely voluntary and you are free to withdraw from the study if you do not wish to continue.

The inconvenience that we could cause you by your participation will be minimal; You will have to answer a battery of questionnaires where information will be collected on some of your personal characteristics (eg self-efficacy, personality), your state of health and quality of life, lifestyles (eg physical activity, diet) and social relationships.

Previously we will ask you to answer some of these questionnaires at home and the rest will be filled out during the face-to-face visit. In addition, we will make an appointment at your health center to carry out a blood test.

At the arranged appointment, a physical examination and the remaining questionnaires will be carried out in person, which will last approximately 1h-1:30h.

We also ask for your authorization to create a database in which, through a random identification number, you will allow your identification data to be completely confidential, your data being anonymized. In this clinical database, the information collected in the completed questionnaires, health status, physical examination (weight, height, BMI) will be collected. In addition, you will be asked for authorization to enter information from the electronic medical record and primary care (OMI) program in this database, which is the one used by primary care professionals to record information related to your health, such as example (previous history , tests, specialist consultations, hospital admissions). With all the information collected, a statistical analysis will be carried out, which allows us to compare the data of the entire population that agrees to participate and observe the relationship between the data and lifestyles, following the end of the study.

In addition, they may be asked to participate voluntarily in small groups of 8-12 people, an exhibition of ideas and opinions will be made following a script of topics, previously piloted by a moderator. The activity in groups will be recorded in audio and video, prior informed consent and will be transcribed literally, anonymizing the identification data of the informants in the transcripts. It will last 1-2 sessions of 30 min.

**SUPPLEMENTARY 1 (Continued)**

***Your Participation and Withdrawal from the Study***

This study has been approved by CEICA Aragón. Your participation in it is voluntary and you can decide to withdraw at any time, even if you have signed the consent and the study is in full swing. Your decision will not affect any health care you receive later. In addition, you have the right to request the study investigators, at any time, and without specifying the reason, the deletion of your data.

No financial compensation is expected for your participation in the study. It will be necessary for an authorized study investigator to be able to access your clinical history to collect data related to the study confidentially and subsequently processed in a completely anonymous manner.

To contact those responsible for the study, you can contact:

-Dra Fátima Méndez López de la Manzanara: Responsible for the project. Telephone 649684202. Email: fatima.lopezmendez@hotmail.com

-Dra Rosa Magallón Botaya: Principal Investigator of the Research Group. Telephone 9976731500. Email: [rosamaga@unizar.es](mailto:rosamaga@unizar.es)

Address: Primary Care Research Unit, Arrabal Health Center Grace Gazulla 16, Zaragoza.

***Contact and monitoring of your health status***

We would also like to be able to contact you in a timely manner to ask you some questions about your health condition and to make appointments for other visits.

If you agree, you may be called to participate in a discussion group regarding the project.

After your participation we will send you a report with the results of some of the explorations carried out and that may be of interest to you.

***Confidentiality***

The entire research team undertakes to carry out the process of data collection, analysis and preparation of the results confidentially and anonymously, in accordance with the provisions of Regulation (EU) 2016/679 of the

European Parliament and the Council of April 27 on Data Protection (RGPD) and the applicable national regulations. The Biomedical Research Law (14/2007) will also be respected, in addition to Law 3/2018 on the protection of personal data and guarantee of digital rights and any other that is applicable. The data will be used exclusively for the purposes of this study.

***Data destination***

The collected data will be stored in a research data file at the IIS Aragon headquarters. With these data, the study researchers will carry out the corresponding analyzes to respond to the objectives of the study

In order to carry out the project that we have presented to you and in accordance with current legal provisions, we request your authorization. Before and after signing this consent, of which you will keep a copy, you can ask the doctors or health personnel responsible for the study anything you deem appropriate.

**SUPPLEMENTARY 1 (Continued)**

**INFORMED CONSENT DOCUMENT**

***Participant statement***

I have been informed by the health professional mentioned below:

- Of the aims and implications of the present study;
- About the process of obtaining, storing and processing personal data;
- That it may be necessary to consult information related to this study from my medical record;
- That compliance with the data protection law is guaranteed. Regulation (EU) 2016/679 of the European Parliament and the Council of April 27 on Data Protection (RGPD);
- That the data obtained is aimed at biomedical research and that the law will be respected to that effect (14/2007);
- That the data obtained will be protected in accordance with Law 3/2018 on the protection of personal data and guarantee of digital rights
- That participation is voluntary and that at any time I can revoke my consent and request the deletion of my personal data and my samples without any repercussion on subsequent health care;
- In addition, I have been able to ask the questions that I have considered appropriate.

After receiving this information: YES NO

- to participate voluntarily in the study: **Exploring the relationship between psychological constructs, lifestyles, and highly prevalent affective-emotional and addictive behaviours in a general population in Primary Health Care.**
- I authorize personnel related to the study to contact me in the future to learn about my health status.
- I would be interested if you contact me to participate in a discussion group in relation to this project.
- That my data can be crossed with an anonymized clinical information platform, to update the data on the appearance of chronic diseases (cardiovascular, cancers, diabetes, lung disease...).
- That my data can be transferred, completely anonymously, to Insignia Health with the aim that this company, owner of the Patient Activation Measure (PAM®) questionnaire, can improve its quality .
- I authorize the project researchers to share the data with other researchers, always guaranteeing confidentiality and current legal and ethical regulations and the objective is related to this study.

***Participant statement***

Name and surname of the participant:… ………………………………………….……………….................... ..............

Signature……………………………… ……. …………. Date…. ……..……………………..

***Statement from the medical health professional that he/she has duly informed the participant***

Name and surname(s) of the health professional:… .............................................. ........................................................... .......

Signature……………………………… ……. ………. Date…. ……..……………………..

***SECTION FOR THE REVOCATION OF CONSENT***

I, ………………………………………………………………………………………………………………… ……. .…, I revoke the consent to participate in this study.

Signature……………………………… ……. ………. Date…. ……..……………………..
